# Bilateral multiple retinal pigment epithelial detachments

**DOI:** 10.1007/s10633-025-10046-x

**Published:** 2025-09-05

**Authors:** Arisa Yoshida, Masayuki Shibuya, Yoshiaki Shimada, Yuro Igawa, Midori Tachibana, Kei Shinoda

**Affiliations:** 1 Department of Ophthalmology, Saitama Medical University Faculty of Medicine, 38 Moro-Hongo Moroyama-machi, Iruma-gun, Saitama, 350-0495 Japan; 2 Department of Ophthalmology, Daiyukai Dai-ichi Hospital, Hagoromo1-6-12, Ichinomiya, Aichi 491-8551 Japan

**Keywords:** Multifocal retinal pigment epithelial detachments, Bilateral retinal pigment epithelial detachments, Large colloid drusen, Cuticular drusen

## Abstract

**Purpose:**

To report a rare case of bilateral idiopathic multifocal retinal pigment epithelial detachments (imfPEDs) and to describe the long-term morphological and functional changes observed over a 16-year follow-up period.

**Methods:**

A 49-year-old woman was diagnosed with imfPEDs based on multimodal imaging, including optical coherence tomography (OCT), fluorescein angiography (FA), and fundus photography. Full-field electroretinograms (ffERGs) and multifocal ERGs (mfERGs) were recorded to assess retinal function. The patient voluntarily discontinued follow-up but returned 16 years later due to cataract progression. Retinal morphology and function were re-evaluated using comparable multimodal imaging and electrophysiological methods.

**Results:**

At the initial visit, multiple bilateral pigment epithelial detachments (PEDs) were identified. OCT showed hyporeflective, dome-shaped PEDs with smooth borders, and ERG responses were within normal limits. Sixteen years later, some PEDs had resolved, others had newly developed or fused, and geographic atrophy was observed, particularly in the peripheral retina. Fundus autofluorescence (FAF), performed in place of FA, revealed hyperautofluorescent PEDs and numerous peripheral hypofluorescent spots. ffERGs remained normal, while mfERGs showed localized attenuation with relatively preserved macular function. These findings were consistent with large colloid drusen and cuticular drusen.

**Conclusion:**

This case demonstrates the slow morphological progression and relative functional preservation in bilateral imfPEDs over 16 years. Comparable multimodal imaging and electrophysiological testing were valuable in monitoring the long-term clinical course and support the classification of this phenotype as a variant of large colloid or cuticular drusen.

**Supplementary Information:**

The online version contains supplementary material available at 10.1007/s10633-025-10046-x.

## Introduction

Bilateral idiopathic multifocal retinal pigment epithelial detachments (imfPEDs) are rare retinal disorders first reported by Gass et al. in 2005 [1]. In their report, Case 1 presented with unilateral vitreous hemorrhage, and after enucleation, histopathological examination revealed multiple serous and hemorrhagic PEDs, sub-retinal pigment epithelial (RPE) neovascularizations, mild focal nongranulomatous choroiditis, fibrocytic proliferation, and a hemorrhagic retinal detachment. The patient was diagnosed with imfPED. Electrophysiological examination of one of these three cases showed normal electro-oculography (EOG) and slightly reduced amplitudes on electroretinograms (ERGs). Several cases have since been reported [2–5], and the prognosis has generally been good. However, there is limited information on the pathophysiological mechanism and the natural history of this retinal disorder. We examined a case that presented with characteristics similar to imfPEDs and were able to obtain the patient’s long-term clinical findings.

Advances in multimodal imaging have provided new understandings in the types and pathology of PEDs [5]. As a result of multimodal imaging, we diagnosed our case as large colloid drusen (LCD) [5, 6]. The optical coherence tomographic (OCT) findings were the most important findings for the diagnosis, but fundus fluorescein angiography (FA) and fundus autofluorescence (FAF) also provided important secondary findings that confirmed the pathology of the PED seen in the OCT images. In addition, we have a long follow-up record of 16 years including electrophysiological test results. We present detailed information on the structure and physiology of the retina and choroid in this case.

## Case report

**Fig. 1:**
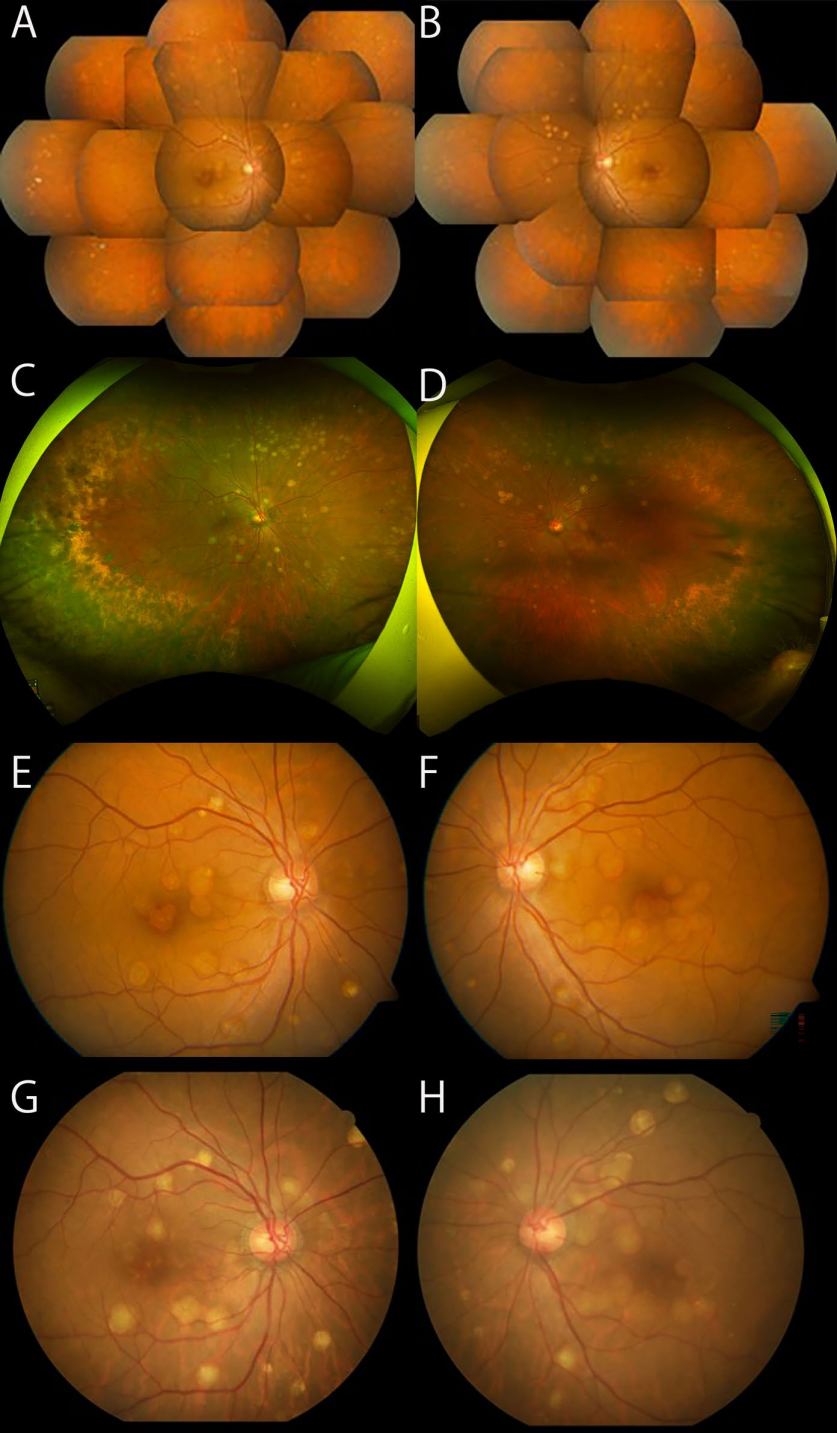
Comparisons of panoramic and widefield posterior fundus photographs with a 16-year interval in a patient diagnosed with bilateral multifocal retinal pigment epithelial detachments. **A** Panoramic fundus photograph of the right eye at the initial visit. The decimal best-corrected visual acuity (BCVA) was 1.0. There are numerous white lesions throughout the retina. The edges of the lesions are smooth, the shape is round or oval, and the size is uniform. **B** Panoramic fundus photograph of the left eye at the initial visit. The decimal BCVA is 1.0. **C** Ultra-widefield fundus photograph of the right eye 16 years after the initial visit. The decimal BCVA is 0.4. Some of the white lesions have disappeared compared to 16 years earlier, but there is no significant change in their size or distribution of the remaining lesions. **D** Ultra-widefield fundus photograph of the left eye 16 years after the initial visit. The BCVA is 0.8. **E** Fundus photograph of the right eye at the initial visit. F Fundus photograph of the left eye at the initial visit. **G** Fundus photograph of the right eye, 16 years after the initial visit. Compared to the findings of A, some white lesions are not present and some new ones are present. **H** Fundus photograph of the left eye 16 years after the initial visit

**Fig. 2:**
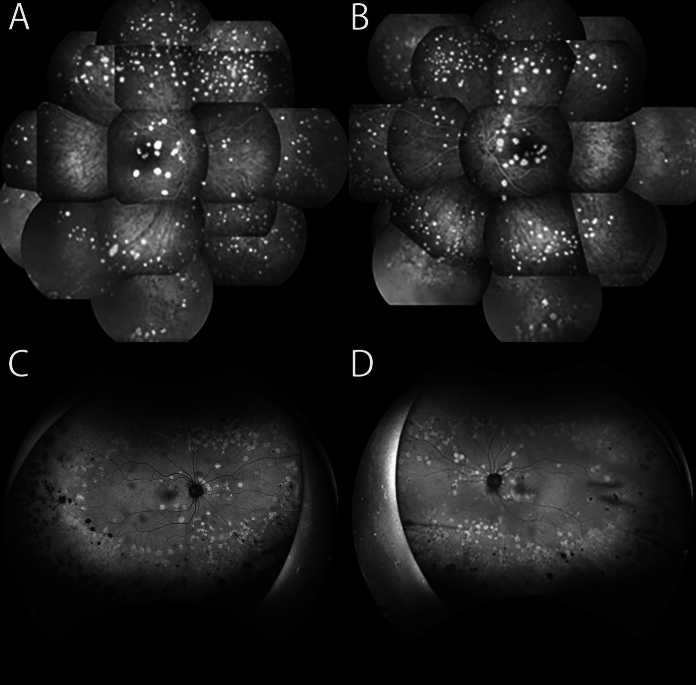
Comparison of the fluorescein fundus angiograms and fundus autofluorescence images with a 16-year interval. **A** Fluorescein fundus angiography of the right eye at initial examination showing multiple hyperfluorescent lesions with smooth edges, round or oval shape, and nearly uniform size. **B** Fluorescein fundus angiogram of the left eye at the initial visit. **C** Fundus autofluorescence (FAF) image shows hyperfluorescence of the right eye 16 years after the initial visit. Compared to the findings of A, some white lesions are not present and some have newly developed. Numerous hypofluorescent spots suggesting geographic atrophy (GA) are observed mainly in the peripheral area. **D** FAF of the left eye 16 years after the initial visit

**Fig. 3:**
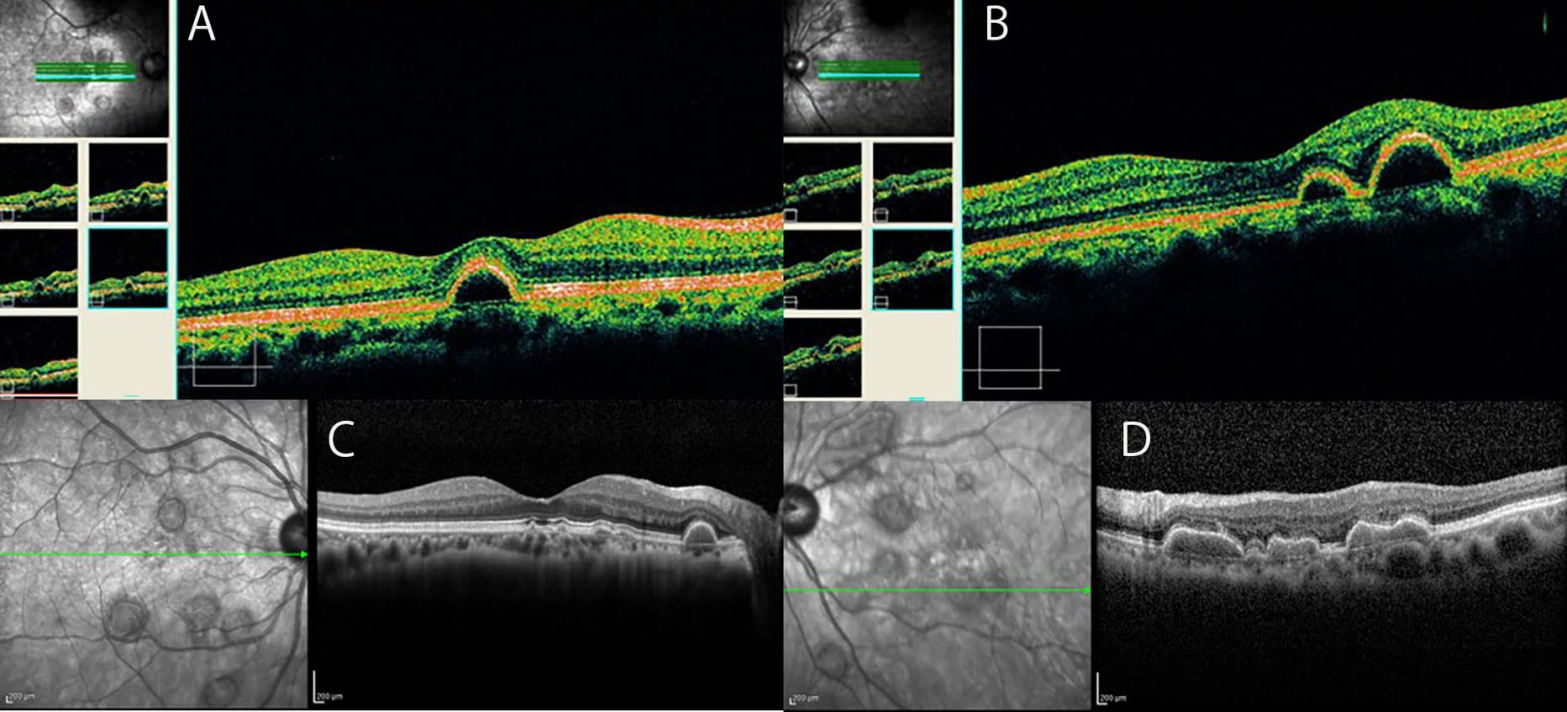
Comparison of optical coherence tomographic images with a 16-year interval. **A** Optical coherence tomographic (OCT) image of the right eye at the initial visit showing pigment epithelial detachments (PED). The PEDs have smooth edges and are oval shape along the z-axis. The inside of the PED is hyporeflective. **B** OCT image of the left eye at the initial visit. **C** OCT image of the right eye 16 years after the initial visit. **D** OCT image of the left eye 16 years after the initial visit showing fused PEDs (red arrow). The inside of the PED is hyperreflectivity which was not found at the initial visit

**Fig. 4:**
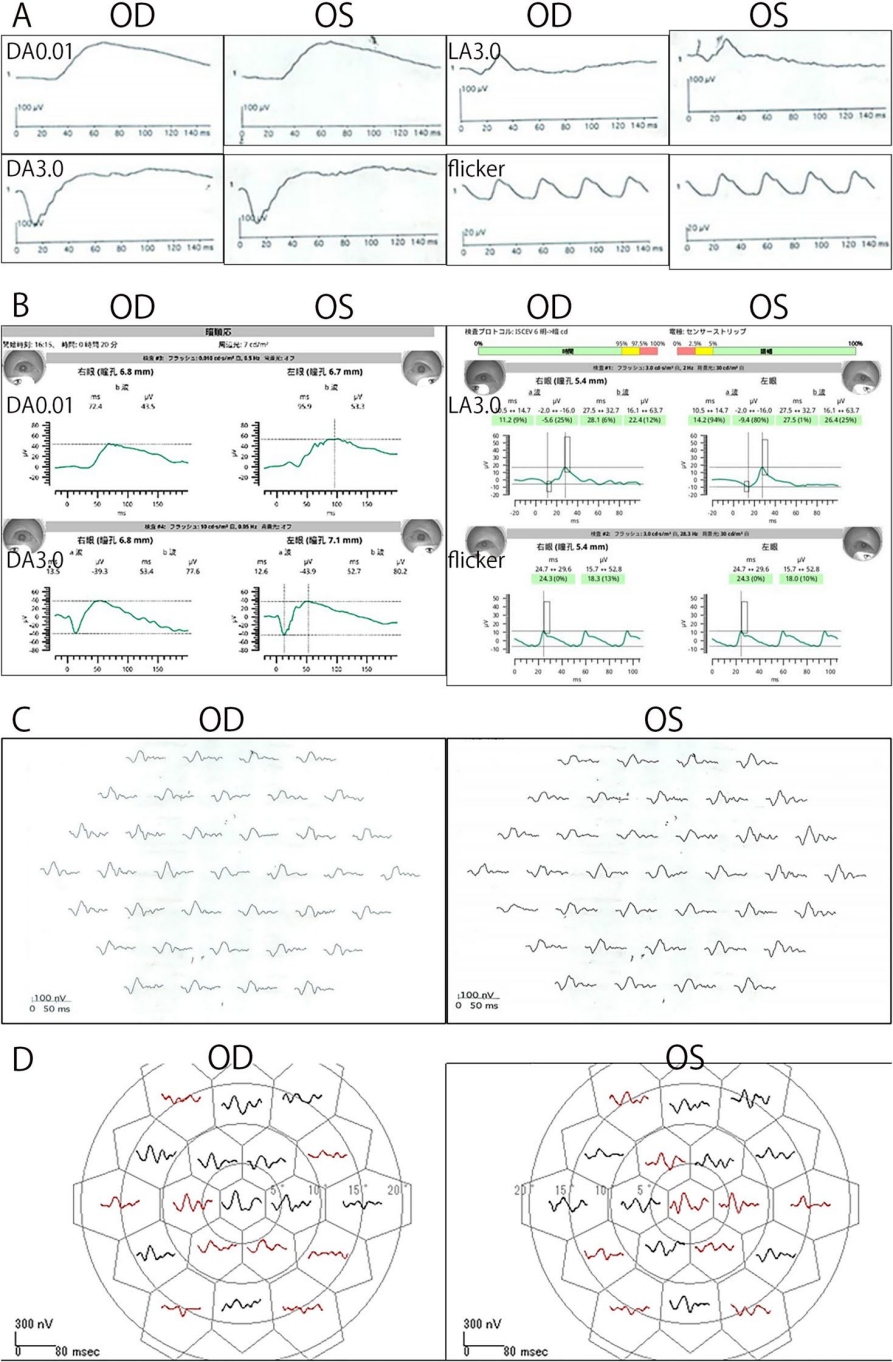
**Comparison of full-field and multifocal electroretinograms (ERGs) recorded with a 16-year interval. A** Full-field ERGs recorded at the initial visit showing normal amplitudes in both eyes. **B** Full-field ERGs recorded 16 years after the initial visit showing no significant changes in the amplitudes in both eyes. **C** mfERGs recorded at the initial visit showing normal macular function in both eyes. **D** mfERGs recorded 16 years after the initial visit showing a slight decrease in the responses in several areas. Otherwise, the response in the macular region was relatively well preserved. The red waveform indicates that the N1-P1 amplitude is attenuated to be outside the normal range ± 2SD

A 49-year-old woman who complained of floaters was examined by a local ophthalmologist and was referred to our hospital with suspected Vogt-Koyanagi-Harada disease in **2008**. There was nothing significant about her family medical or medication history although the patient's parents were first cousins. The decimal best-corrected visual acuity (BCVA) was 1.0 in both eyes, and the intraocular pressure (IOP) was 18 mmHg in the right eye and 20 mmHg in the left eye. No significant findings were observed in the anterior segment, but ophthalmoscopy showed multiple white lesions **(**Figs. 1**)** corresponding to retinal PEDs in the OCT images. FA also showed uniform hyperfluorescence of the white lesions **(**Fig. 2**).** Spectral domain OCT (SD-OCT; Cirrus HD-OCT, Carl Zeiss Meditec) showed that the white lesions were uniformly hyporeflective **(**Fig. 3**)** in both eyes. The full-field ERGs (ffERGs, LE-4000, Tomey Corporation, Nagoya) and multifocal ERGs (mfERGs, VERIS™ Science 5.1.12 system, EDI: Electro-Diagnostic Imaging, Milpitas, CA, USA) were normal **(**Fig. 4**)**. The patient was diagnosed with imfPED based on the findings, and she was followed without any interventions. She voluntarily stopped visiting our hospital in 2008 + **1**.

Sixteen years later, at age 65, the patient was referred again for surgery for a cataract in her left eye. At our examination, her decimal BCVA was 0.8 in the right eye and 0.4 in the left eye, the IOP was 17 mmHg in both eyes, and moderate cataracts were detected in both eyes. The visual acuity in the left eye improved to 0.9 after cataract surgery. Ultra-widefield fundus ophthalmoscopy (Optos^Ⓡ^California, Nikon Solutions Co., Ltd., Tokyo) showed that some of the PEDs had disappeared compared to 16 years earlier, but there was no significant change in the size or distribution of the remaining white lesions **(**Figs. 1**)**. Pigmentation and chorioretinal atrophy were observed in the peripheral retina. FAF showed hyperfluorescence of the PEDs, and numerous hypofluorescent spots were detected mainly in the periphery **(**Fig. 2**)**. SD-OCT (SPECTRALIS HRA 2, Heidelberg Engineering, Heidelberg, Germany) showed that some of the PEDs had resolved, or partially fused **(**Fig. 3**)**, and some had newly developed. Some of the PEDs had moderate internal reflection.

The ffERGs recorded with the RETeval® system (LKC Technologies, Gaithersburg, USA) using skin electrodes were normal **(**Fig. 4**)**. Although the mfERGs (LE-4000 mfERG ver.3.27: TOMEY, Nagoya, Japan) were mildly decreased in several areas, the responses in the macular region were relatively well preserved **(**Fig. 4**)**.　　　　　　　　　　　　　　　　　　　　　　　　　　　　　　　　　　

## Discussion

The visual prognosis of imfPED is generally good based on the BCVAs, the Amsler chart scores, and electrophysiological findings (Supplemental Table) [1–4], but hemorrhagic PEDs and macular neovascularization (MNV) can develop.^1^ The location of the PEDs varies depending on the case, and may involve only the macula, or outside the vascular arcade in the posterior pole, or up to the equator. They are usually bilaterally symmetrical. None of the cases had a known family history or consanguineous marriage. Our patient was relatively young when diagnosed, and we were able to observe the long-term progression of the imfPEDs. After the longest follow-up period of 16 years, some of the PEDs had disappeared or fused, and RPE atrophy occurred in the periphery. However, the visual acuity, ffERGs, and mfERGs indicated good retinal physiology.

The lesions were scattered throughout the retina and were similar to large colloidal drusen (LCD) in that they had a yellowish-white appearance on ophthalmoscopy and had a hyperreflective lumen in the OCT images [6, 7]. The imfPEDs are thought to be the same clinical entity as LCD [6, 7] and both are subtypes of cuticular drusen because of having similar findings in multimodal images [5, 8, 9]. Typically, the lesions in both disorders are hyperfluorescent in FA and hypofluorescent in the area corresponding to the apex of the drusen in the FAF images. LCD is a subgroup of early onset drusen (EOD) that are present in young patients [10, 11]. The LCD are large, 200–300 μm, bilateral, yellowish, and scattered in the macula and peripheral retina [12]. Most LCD have a convex shape with medium and homogeneous internal reflectivity in the OCT images [13]. The LCD are grouped with the EOD, and both have been considered to be benign since the original description [10]. However, it has been reported recently that LCDs may develop macular neovascular membranes (MNV) [9, 11, 14] which is in line with the MNV in a case of imfPED reported by Gass et al.^1^

The reflectivity of cuticular drusen in OCT images is reported to be highly variable, with iso-, hypo-, and hyperreflective areas [5]. In FAF, they are typically characterized by a hypoautofluorescent center with hyperautofluorescent margins [5]. They progress to geographic atrophy (GA) more frequently in patients older than 60 years of age [5].

The white lesions in our case had smooth edges and were round or oval with an almost uniform size. The corresponding PEDs were round or oval spatially along the z-axis in the OCT images. Although the inner reflectivity was low at the initial visit, it became higher in some PEDs 16 years later. The FAF at a recent visit showed homogeneous hyperfluorescence including that at the apex, and there was diffuse geographic atrophy (GA) especially in the peripheral retina. Interestingly, the GA was less likely to occur in the posterior pole than at the periphery. These findings are generally consistent with LCD and cuticular drusen, although some are atypical.

Nearly normal ffERG and mfERG responses may help exclude inherited dystrophies or other diffuse retinal diseases, even if they do not confirm a specific diagnosis. Although the ffERGs and mfERGs were recorded 16 years apart, the procedures used conformed to the ISCEV standards for both recordings. However, they were recorded using different instruments and the most recent recordings were made using skin electrodes, so caution is required when comparing them. The mfERGs were initially normal and mildly reduced at the follow-up examinations. The areas of ​​reduction did not necessarily coincide with the PED, and the cause is unknown. We plan to continue to perform fundus imaging as well as electrophysiological evaluations in this patient.

The earlier reported cases of bilateral multifocal PED had a good prognosis except for one case with the longest follow-up of 4 years and 7 months in which neovascularization occurred [1–4]. Interestingly, panel-based genetic testing was performed in one case, but the results were negative [3]. In our case, the visual functions, including the visual acuity and ERGs, were mainly normal over the longest follow-up period of 16 years. In addition, the multifocal PED either disappeared, newly developed, or some fused, resulting in GA mainly in the periphery. Multimodal imaging such as OCT and FAF, and ERGs were helpful in evaluating the changes in the appearance of the fundus.

Alhumaid et al. stated that panel-based genetic testing did not reveal any pathogenic mutations in their case 2 [3]. We have not performed genetic testing, and it may be warranted in the future, given the presence of consanguinity.

## Conclusion

In conclusion, the detailed findings of a rare case of imfPED with a 16-year follow-up period are presented. We conclude that this is a subtype of LCD with cuticular drusen. The multifocal PEDs changed and GA developed mainly in the periphery. Multimodal imaging and ERGs were helpful to evaluate the long-term clinical course of LCD.

## Supplementary Information

Below is the link to the electronic supplementary material.

Supplementary file1 (XLSX 13 KB)

## Declarations

### Statement on the welfare of animals

This article does not contain any studies with animals performed by any of the authors.
